# *FAM83B* is involved in thyroid cancer cell differentiation and migration

**DOI:** 10.1038/s41598-022-12553-2

**Published:** 2022-05-21

**Authors:** Valentina Cirello, Elisa Stellaria Grassi, Gabriele Pogliaghi, Viola Ghiandai, Laura Ermellino, Marina Muzza, Giacomo Gazzano, Luca Persani, Carla Colombo, Laura Fugazzola

**Affiliations:** 1 Laboratory of Endocrine and Metabolic Research, Istituto Auxologico Italiano IRCCS, Milan, Italy; 2 Department of Medical Biotechnology and Translational Medicine, University of Milan, Milan, Italy; 3 Department of Pathophysiology and Transplantation, University of Milan, P.le Brescia 20, 20149 Milan, Italy; 4 Pathology Unit, Istituto Auxologico Italiano IRCCS, Milan, Italy; 5 Division of Endocrine and Metabolic Diseases, Istituto Auxologico Italiano IRCCS, Milan, Italy

**Keywords:** Cancer, Endocrinology

## Abstract

*FAM83B* has been recently identified as an oncogene, but its role in thyroid cancers (TC) is still unclear. We examined the expression of *FAM83B* and its possible involvement in cell migration and differentiation, in neoplastic/normal thyroid tissues and in TC human cell lines. *FAM83B* expression in TC varies according to the tumor histotype, being significantly downregulated in more aggressive and metastatic tissues. *FAM83B* levels in cell lines recapitulate patients’ samples variations, and its total and cytoplasmic levels decrease upon the induction of migration, together with an increase in its nuclear localization. Similar variations were detected in the primary tumor and in the metastatic tissues from a follicular TC. *FAM83B* knock down experiments confirmed its role in thyroid differentiation and cell migration, as demonstrated by the reduction of markers of thyroid differentiation and the increase of the mesenchymal marker vimentin. Moreover, the silencing of *FAM83B* significantly increased cells migration abilities, while not affecting the oncogenic RAS/MAPK/PI3K pathways. Our data indicate for the first time a role for FAM83B in TC cell differentiation and migration. Its expression is reduced in dedifferentiated tumors and its nuclear re-localization could favour distant migration, suggesting that *FAM83B* should be considered a possible diagnostic and prognostic biomarker.

## Introduction

In the last decade, the *FAM83B* gene (Family with sequence similarity 83, member B) has been proposed as an oncogene capable of driving the transformation of immortalized human mammary epithelial cells (HMEC), similar to mutant *RAS*^1^. FAM83B is one of the eight members of a protein family (FAM83A-H) characterized by a highly conserved domain of unknown function (DUF1669), which is necessary and sufficient to drive transformation^2^. Recent studies have indicated that the DUF1669 domain mediates the interaction with different isoforms of the casein kinase 1 (CK1) family of Ser/Thr protein kinases involved in diverse cellular processes such as membrane trafficking, cytoskeleton maintenance, DNA replication, DNA damage response and Wnt signaling^3–5^. Each FAM83 family member has a unique C terminus of variable length with distinct biological functions, being FAM83B involved in the activation of MAPK, PI3K-AKT-mTOR, Wnt/β-catenin signaling pathways and EGFR-PDL1 axis, thus promoting cell proliferation, anchorage-independent growth, and tumorigenicity^1,2,6–8^. *FAM83B* mRNA levels are reported to be significantly increased in some cancer subtypes and has been shown to be a poor prognostic factor for breast, lung and pancreas cancers^1,9–14^. Both *FAM83B* and other *FAM83* family members (i.e. *FAM83A*, *FAM83D*, *FAM83G*, and *FAM83H*) have been reported to be involved in the regulation of cell migration in different tumors (osteosarcoma, endometrial, colon, cervical and gastric cancer)^12,15–19^. On the other hand, it has been very recently reported that ovarian cancer patients with low *FAM83B* levels have shorter survival time and show cisplatin resistance^8^, while high expression of *FAM83B* correlates with prolonged progression-free interval in triple negative breast cancer^20^.

As far as thyroid cancer (TC) concerns, *FAM83B*, as well as another family member-*FAM83F*^21^, was found to be overexpressed in a limited series of papillary TCs, but data on larger series are lacking^1,22^. To get more insights into this oncogene, barely studied despite its potential role in TC progression, we examined the expression status, the subcellular localization and the role in migration of *FAM83B* in TCs and normal thyroid tissues, and in TC cell lines. The correlation between *FAM83B* expression and the clinico-pathological features and prognosis of patients was studied, too. Our results suggest for the first time a role for FAM83B in thyroid cell migration and differentiation, as demonstrated by the reduction of markers of thyroid differentiation and the increase of the mesenchymal marker vimentin, and by the increase in cells migration abilities upon *FAM83B* silencing.

## Results

### FAM83B mRNA levels in normal and neoplastic thyroid tissue: downregulation and thyroid cell dedifferentiation

**Figure 1:**
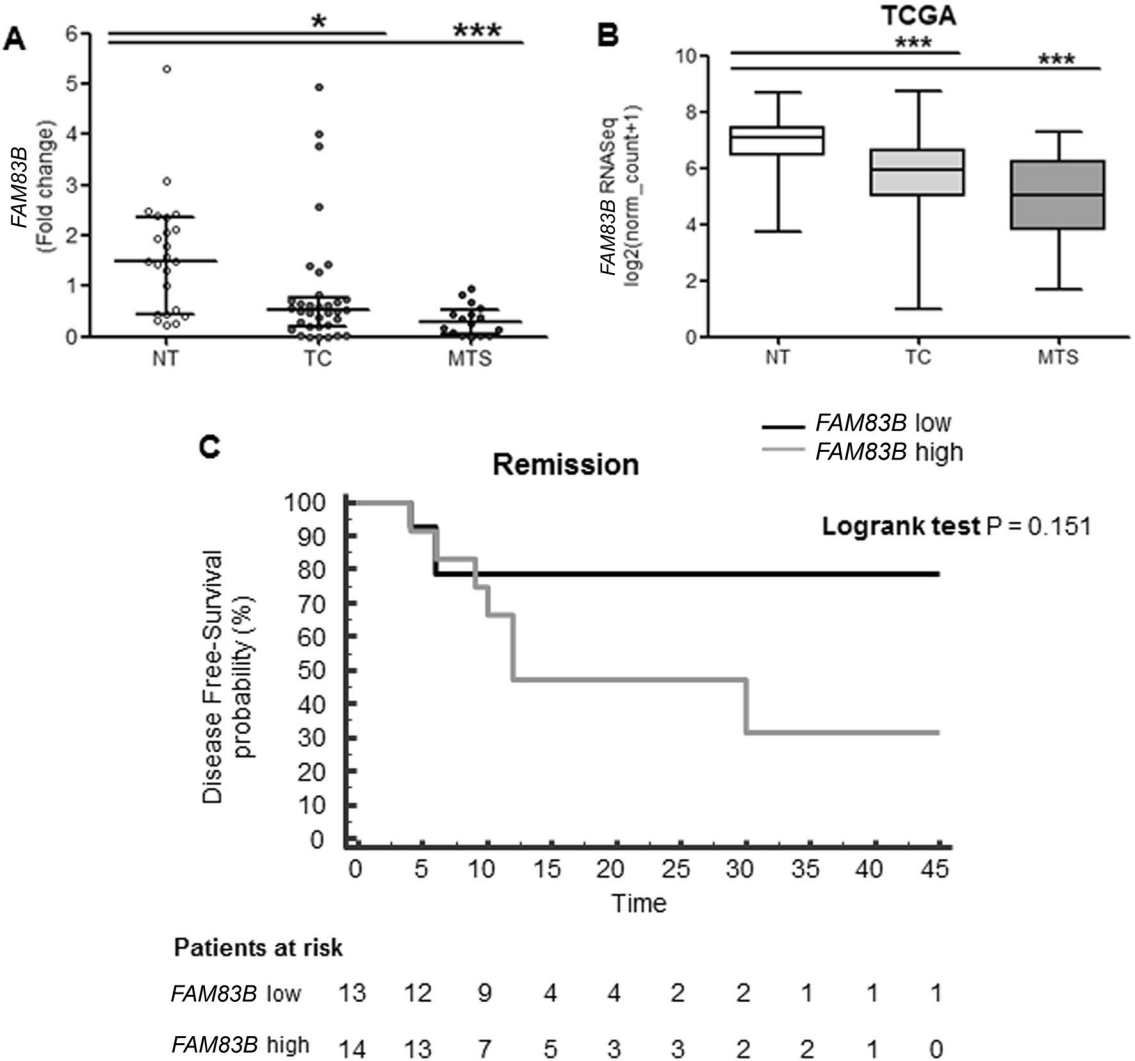
*FAM83B* mRNA levels in normal and neoplastic thyroid tissues and correlation with disease-free survival (DFS) survival. (**A**) *FAM83B* levels evaluated in our cohort of 39 patients [Normal Thyroid (NT) n = 24, Thyroid Cancer (TC) n = 34 and Metastasis (MTS) n = 16]. For some patients NT, TC and MTS paired samples were available. Data are shown as dot-plot with median and interquartile range. (**B**) *FAM83B* levels in TCGA THCA database. Thyroid Cancer (TC) n = 501, Normal Thyroid (NT) n = 58 and Metastasis (MTS) n = 8. Data are shown as box and whiskers (min to max). (**C**) Kaplan–Meier analysis of DFS in patients according to *FAM83B* expression. Patients were divided in *FAM83B*-high and *FAM83B*-low based on *FAM83B* TC group median value (0.517). Survival curves were generated using the Kaplan–Meier method and the log-rank test was used to evaluate the statistical significance of differences; patients were censored at the time of remission or, in the case of no remission, at the time of the last clinical visit.

*FAM83B* mRNA levels were initially evaluated in 24 normal thyroid (NT), 34 thyroid cancer (TC) and 16 metastatic (MTS) tissue samples. *FAM83B* mRNA was significantly downregulated in TC and MTS samples compared to NT (p < 0.05 and p < 0.001, respectively) (Fig. 1A). Similar data were obtained analyzing data extracted from the TCGA THCA database (Fig. 1B). No significant differences were found in the clinico-pathological characteristics between tumors with high or low *FAM83B* levels, with the exception of the histotype which was more frequently PDTC/ATC in the *FAM83B* low group (38 vs 7%, P = 0.02). Interestingly, though not reaching the statistical significance, there was a clear tendency towards a higher frequency of distant metastatization in the *FAM83B* low group (64 vs 21%, P = 0.18) (Supplementary Table 1).

Consistently, patients with low *FAM83B* levels tended to have a shorter disease-free survival (DFS) rate than those with high *FAM83B* levels (Log rank 0.151) (Fig. 1C). When considering PTCs separately (n = 20), DFS was not significantly different between cases with low or high FAM83B expression (P = 0.224), possibly due to the low statistical power of the sample.

### FAM83B mRNA expression profile in normal thyroid tissue, primary thyroid cancers and metastases

**Figure 2:**
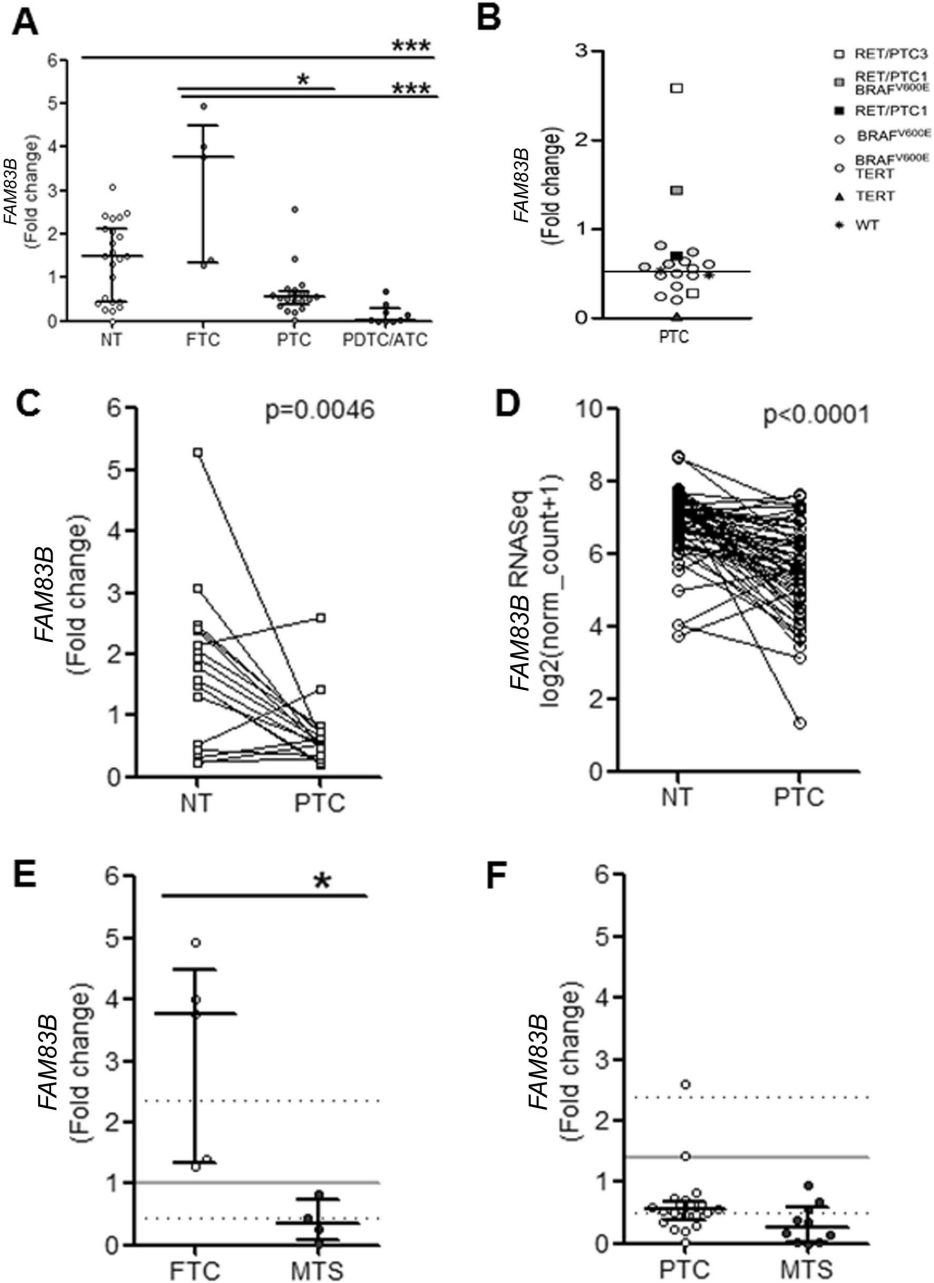
*FAM83B* levels are significantly different among normal thyroid tissue, primary thyroid tumors and metastases. (**A**) *FAM83B* levels evaluated in our cohort of TC patients (Normal Thyroid (NT) n = 24, Follicular Thyroid Cancer (FTC) n = 5, Papillary Thyroid Cancer (PTC) n = 20, Poorly differentiated and Anaplastic Thyroid Cancer (PDTC/ATC) n = 9. For some patients normal and cancer tissue paired samples were available. Data are shown as dot-plot with median and interquartile range. (**B**) *FAM83B* levels in PTC samples grouped by molecular profile. Data are shown as dot-plot with median. White square: *RET/PTC3*, Gray square: *RET/PTC1* and *BRAFV600E*, Black square: *RET/PTC1*, White circle: *BRAFV600E*, Gray circle: *BRAFV600E* and *TERT*, Black triangle: *TERT*, Asterisk: Wild type (WT). (**C**) *FAM83B* levels in paired NT and PTC samples available in our cohort (n = 18). Data are shown as paired aligned dot-plot. (**D**) *FAM83B* levels in paired NT and PTC samples available in TCGA THCA database (n = 58). Data are shown as paired aligned dot-plot. (**E**) *FAM83B* levels in unpaired FTC (n = 5) and FTC-derived MTS (n = 4). Data are shown as dot-plot with median and interquartile range. Gray lines represent the median (continuous line) and interquartile range (dotted lines) of the relative Normal Thyroid samples. (**F**) *FAM83B* levels in unpaired PTC (n = 20) and PTC-derived MTS (n = 10). Data are shown as dot-plot with median and interquartile range. Gray lines represent the median (continuous line) and interquartile range (dotted lines) of the relative Normal Thyroid samples. Statistical analysis: (**A**) Kruskal–Wallis test followed by Dunn's Multiple Comparison Test; (**C**,**D**) Wilcoxon signed rank test (matched pairs); (**E**,**F**) Mann–Whitney test.

Since a huge *FAM83B* expression variability was observed in TC group, we decided to analyze separately the 34 tumors with different tumor histotypes as follows: 5 follicular thyroid cancers (FTC), 20 papillary thyroid cancers (PTC), and 9 poorly differentiated/anaplastic thyroid cancers (PDTC/ATC). *FAM83B* mRNA was higher in FTCs compared to either NTs, or PTCs, or PDTC/ATCs (p = ns, p < 0.05 and p < 0.001, respectively). On the other hand, *FAM83B* levels were constantly lower in PDTC/ATCs with respect to NTs (p < 0.001). PTCs were downregulated with respect to NTs, when considering the median levels of *FAM83B* expression, but a huge variability was recorded (Fig. 2A). Immunohistochemistry experiments showed that FAM83B protein levels parallel mRNA levels, with a positive cytoplasmatic staining being detected in normal thyroid tissue and in FTC samples, but not in PTC and PDTC cases (Supplementary Fig. 1). Interestingly, among PTCs, we found that the 2 *RET/PTC1* mutated cases and 1 out of 2 *RET/PTC3* cases had expression levels higher than the median, while *BRAF* mutated and wild type cases were at the median or below the median levels (Fig. 2B). For some patients we had the opportunity to evaluate the *FAM83B* expression in paired PTC/NT tissues and we found that *FAM83B* mRNA is significantly lower in tumors than in contralateral normal thyroid tissues (p = 0.0046) (Fig. 2C), consistent with data extracted from the TCGA THCA database (Fig. 2D). Considering FTC and PTC tissues and their unpaired metastases (FTC- and PTC-derived MTS), we observed that *FAM83B* mRNA level were significantly lower in FTC-derived MTS compared to primary FTCs (p < 0.05), whereas the decrease observed between PTCs and PTC-derived MTS was not significant (Fig. 2E,F).

### FAM83B levels in human thyroid cell lines recapitulate patient samples variations and significantly change upon cell migration

**Figure 3:**
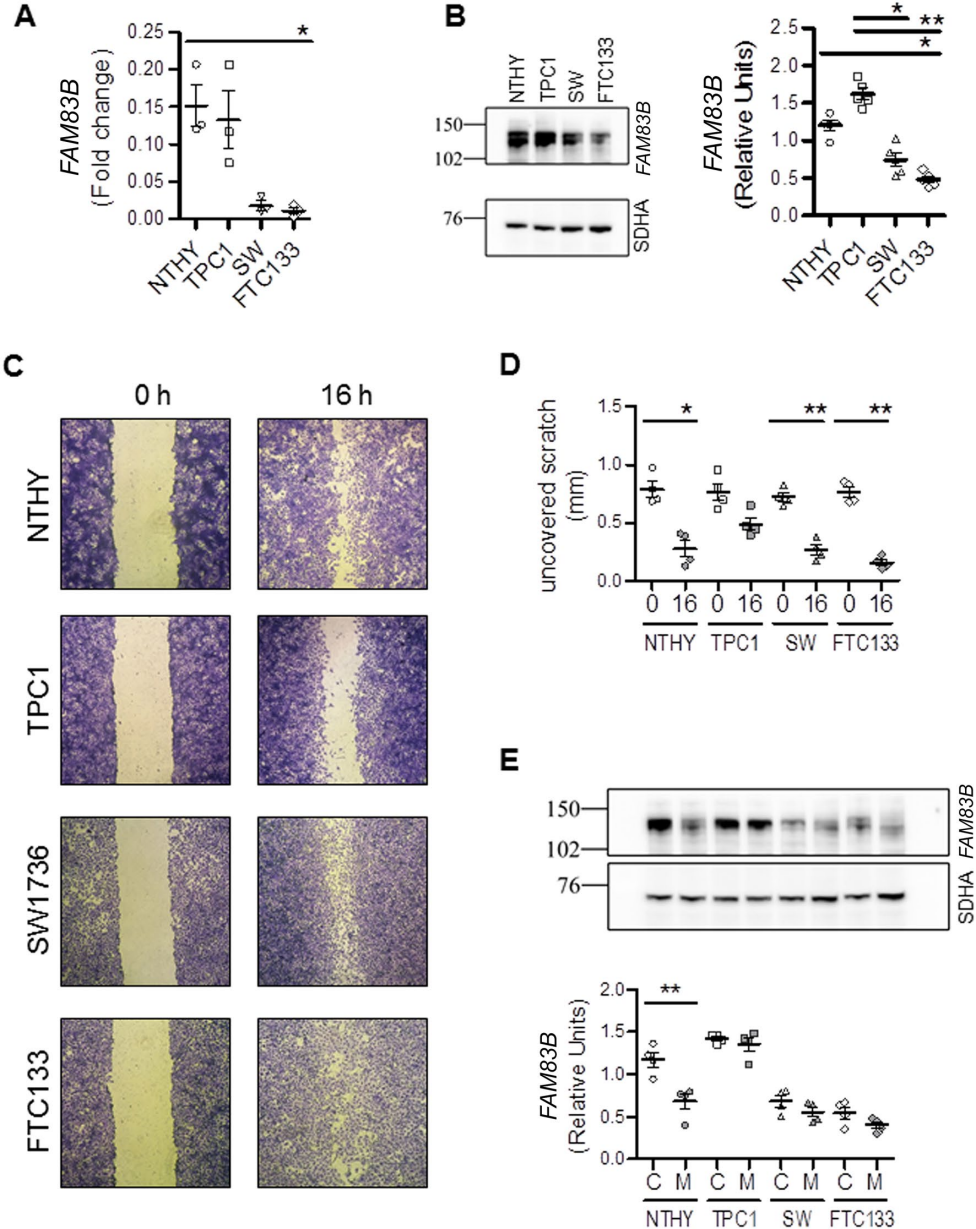
*FAM83B* levels in human thyroid cell lines recapitulate patient samples variations and are influenced by cell migration. (**A**) *FAM83B* mRNA levels in human cell lines derived from normal thyroid (NTHY-ORI), PTC (TPC1), ATC (SW1736) and FTC metastasis (FTC133). n = 3 for all cell lines. (**B**) Representative images and densitometric analysis of western blots showing FAM83B expression levels in the different human thyroid cell lines. SDHA was used as loading control. Data are shown as dot-plot with mean ± SEM. n = 5 for all cell lines. Full-length blot images are shown in Supplementary Fig. 4. (**C**) Representative images of a wound-healing assay showing the migration ability of the different human thyroid cell lines at the indicated times. (**D**) Quantification of uncovered scratch length of the wound healing assays. n = 4 for all cell lines. (**E**) Representative images and densitometric analysis of western blots showing FAM83B expression levels in bulk (C) and migrating (M) cells. SDHA was used as loading control. Data are shown as dot-plot with mean ± SEM. n = 4 for all cell lines. Full-length blot images are shown in Supplementary Fig. 4. Statistical analysis: (**A–E**) Kruskal–Wallis test followed by Dunn's Multiple Comparison Test.

In order to get more insights into *FAM83B* mRNA levels variations found in the different thyroid cancer subtypes, we investigated its expression in cell lines derived from healthy and neoplastic thyroid tissues. Preliminary western blot experiment performed on 8 different cell lines derived from healthy tissue (NTHY-ORI), PTCs (TPC1 and K1), PDTC/ATC (SW579 and SW1736) and metastasis (HTCC3 and FTC133) showed that FAM83B levels in the different cell lines replicate those observed in patients of the present series and in the TCGA cohort (Supplementary Fig. 2). In this context, paralleling the above reported findings in tissues, *FAM83B* expression was lower in K1 cells, which harbor a *BRAF* mutation, than in TPC1 cells, which carry a *RET/PTC* fusion. We selected one representative cell line for each group for the following experiments. Real-time qRT-PCR experiments showed a variability in *FAM83B* mRNA levels among the different cell lines. In particular, NTHY-ORI (derived from normal tissue) and TPC1 (derived from PTC with RET/PTC1 fusion) cells expressed high levels of *FAM83B*, while SW1736 (derived from ATC) and FTC133 (derived from an FTC metastasis) expressed low levels (Fig. 3A). Western blot experiments revealed that FAM83B protein levels vary accordingly to the mRNA ones (Fig. 3B). These cells lines are known to harbor different migration ability and sensitivity to contact inhibition. Namely, NTHY-ORI have high motility in a non-confluent state and a low motility when confluent, TPC1 have a low motility either in non-confluent or confluent state, whereas SW1736 and FTC133 always harbor high motility consistent with their aggressive features^23–29^. Thus, we investigated if the differences detected in *FAM83B* expression were due to differences in cell movement and migration. Scratch assays demonstrated that all cell lines were able to migrate with similar abilities, with the exception of TPC1 cells, that showed low motility (Fig. 3C,D). Moreover, Western blot experiments showed that FAM83B protein levels decreased in all migration induced cells, again with the exception of TPC1 cells (Fig. 3E).

### FAM83B subcellular localization varies during cell migration

**Figure 4:**
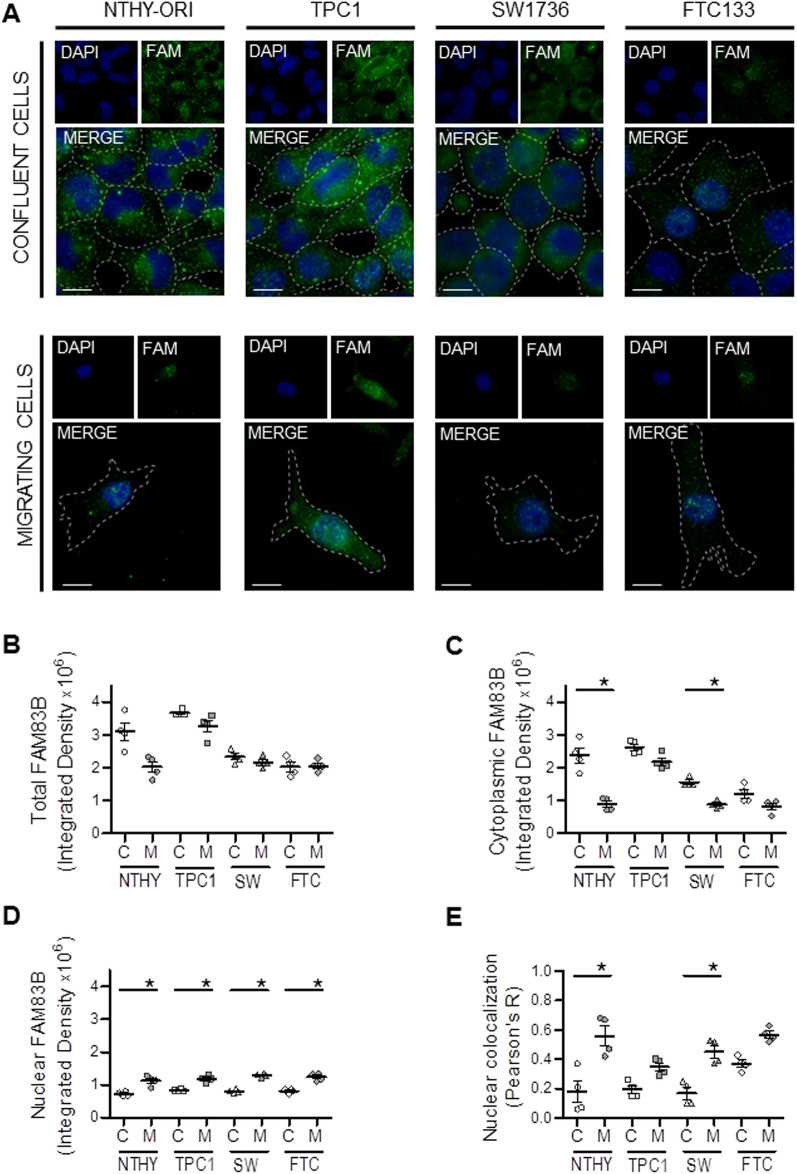
FAM83B subcellular localization significantly varies during cell migration. (**A**) Representative images of immunofluorescence experiments showing confluent and migrating cell lines. Dashed lines highlight cell morphology as determined by WGA staining (see Supplementary Fig. 3). Scalebars 10 µm. Four experiments were done for each cell line. The total number of cells analyzed was 110 for NTHY-ORI 3-1 (57 confluent and 53 migrating), 136 for TPC1 (66 migrating and 70 confluent), 135 for SW1736 (64 confluent and 71 migrating), 97 for FTC133 (56 confluent and 41 migrating). Total (**B**), Cytoplasmic (**C**) and nuclear (**D**) FAM83B levels quantified as Raw Integrated Density in the same experiments as in (**A**). (**E**) Nuclear co-localization with DAPI, with Pearson’s R coefficient. Statistical analysis: (**B–E**) Kruskal–Wallis test followed by Dunn's Multiple Comparison Test.

We then investigated the subcellular localization of FAM83B in quiescent and migrating cells by direct immunofluorescence. All cell lines had a diffuse cytoplasmic signal and, in agreement with the above reported Western blot data, the signal was stronger in quiescent TPC1 and NTHY-ORI, while it was extremely low in SW1736 and FTC133 cells (Fig. 4A,B, Supplementary Fig. 3). Interestingly, the induction of cell migration is accompanied by a reduction in the FAM83B cytoplasmic signal (Fig. 4A,C, Supplementary Fig. 3), strictly correlated to the ability to move. In particular, the mobility and the lack of contact inhibition associated to a significant decrease in FAM83B positivity in NTHY-ORI and in SW1736 cells. On the other hand, not significant reductions were seen either in TPC1 cells (due to the above-mentioned low motility) or in and FTC133 (which already displayed very low positivity due to their high motility). The decrease in cytoplasmic FAM83B was accompanied by an increase in the nuclear localization in all migrating cells (Fig. 4D,E).

**Figure 5:**
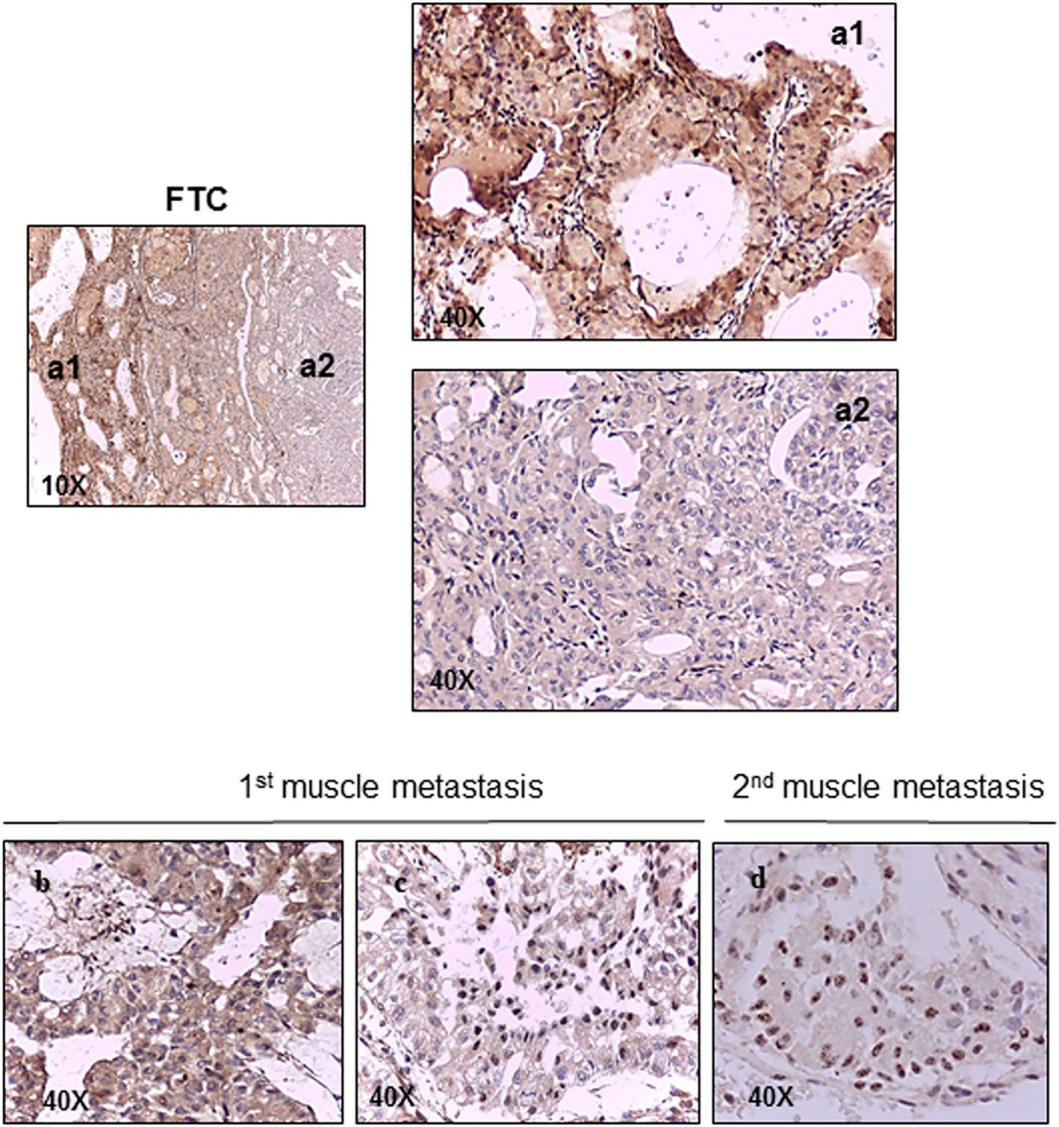
FAM83B expression in thyroid paraffin-embedded tissues from a patient affected with aggressive FTC. A positive cytoplasmatic FAM83B staining was observed in the primary tumor (**a1**) with a progressive lost by going towards the more undifferentiated area (**a2**) (magnification ×10). The first muscle metastasis analyzed showed a positive cytoplasmic staining in the center and a nuclear expression in the peripheral area (**b**,**c**) (magnification ×40). A homogeneous nuclear staining for FAM83B was found in the second muscle metastasis (**d**) (magnification ×40).

To confirm at the tissue level the results on migration and subcellular localization, we analyzed FFPE samples from a patient affected with metastatic and aggressive FTC. In particular, we obtained the primary tumor and two neck muscle metastases obtained from different surgeries at 6.5 months and 1.5 year after thyroidectomy. In the primary tissue, we observed a positive cytoplasmic FAM83B staining which was progressively lost moving towards the more undifferentiated area (Fig. 5). The first local muscle metastasis showed a positive cytoplasmic staining in the center, and a nuclear expression in the peripheral area, while the second local muscle metastasis showed a homogeneous positive nuclear staining for FAM83B (Fig. 5).

### FAM83B silencing reveals its role in thyroid cancer cell differentiation and migration

**Figure 6:**
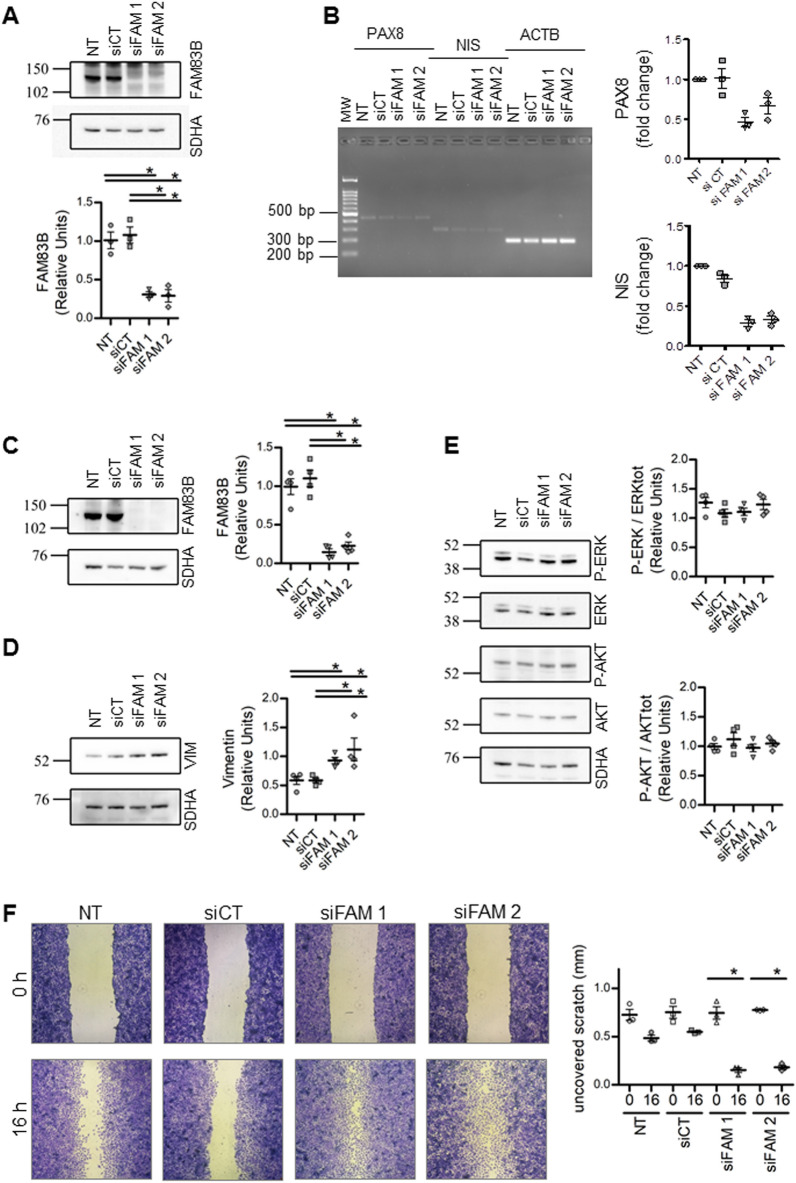
FAM83B silencing reveals its role in thyroid cells differentiation and migration abilities. (**A**) Representative images and densitometric analysis of Western blots showing FAM83B expression levels in NTHY-ORI non-transfected (NT) or transfected with siRNA scramble as control (siCT) and against FAM83B (siFAM 1 and siFAM 2). SDHA was used as loading control. Data are shown as dot-plot with mean ± SEM. n = 3 for all cell lines. Full-length blot images are shown in Supplementary Fig. 5. (**B**) Representative images and densitometric analysis of semiquantitative RT-PCR amplicons run on 2% agarose gel showing *PAX8* and *NIS* expression levels in NTHY-ORI non-transfected (NT) or transfected with siRNA scramble as control (siCT) and against FAM83B (siFAM 1 and siFAM 2). *ACTB* was used as loading control. Data are shown as dot-plot with mean ± SEM. n = 3 for all cell lines. Full-length agarose gel images are shown in Supplementary Fig. 5. (**C**) Representative images and densitometric analysis of western blots showing FAM83B expression levels in TPC1 non transfected (NT) or transfected with siRNA mock as control (siCT) and against FAM83B (siFAM 1 and siFAM 2). SDHA was used as loading control. Data are shown as dot-plot with mean ± SEM. n = 4 for all cell lines. Full-length blot images are shown in Supplementary Fig. 5. (**D**) Representative images and densitometric analysis of western blots showing vimentin (VIM) expression levels in TPC1 non transfected (NT) or transfected with siRNA scramble as control (siCT) and against FAM83B (siFAM 1 and siFAM 2). SDHA was used as loading control. Data are shown as dot-plot with mean ± SEM. n = 4 for all cell lines. Full-length blot images are shown in Supplementary Fig. 6. (**E**) Representative images and densitometric analysis of western blots showing phospho-ERK1/2 (P-ERK), total ERK1/2 (ERK), phosphor-AKT (P-AKT) and total AKT (AKT) expression levels in TPC1 non transfected (NT) or transfected with siRNA mock as control (siCT) and against FAM83B (siFAM 1 and siFAM 2). SDHA was used as loading control. Data are shown as dot-plot with mean ± SEM. n = 4 for all cell lines. Full-length blot images are shown in Supplementary Fig. 7. (**F**) Representative images of a wound-healing and correspective quantification of uncovered scratch length in TPC1 non transfected (NT) or transfected with siRNA mock as control (siCT) and against FAM83B (siFAM 1 and siFAM 2). n = 4 for all conditions. Statistical analysis: (**A**–**D**), Kruskal–Wallis test followed by Dunn's Multiple Comparison Test.

To better elucidate the role of FAM83B in the maintenance of thyroid cell differentiation and in cell migration, we performed silencing experiments using two different siRNAs^11^ in the two cell lines, NTHY-ORI and TPC1, which expressed high *FAM83B* levels. We found that the knock down of *FAM83B* significantly affect normal and tumor thyroid cell differentiation, inducing in NTHY-ORI a reduction in the expression of the thyroid marker genes *PAX8* and *NIS* (Fig. 6A,B), and in TPC1 cells a significant increase in the expression of the mesenchimal marker vimentin (Fig. 6C,D). On the other hand, the silencing of *FAM83B* did not affect the oncogenic RAS/MAPK/PI3K pathways, as the phosphorylation levels of two of the main downstream effectors, ERK and AKT, were unchanged (Fig. 6E). Finally, the *FAM83B* silencing significantly increased the migration abilities of TPC1 cells, that reached levels similar to those observed in SW1736 and FTC133 cells (Fig. 6F).

## Discussion

*FAM83B* has been recently identified as an oncogene involved in the development and progression of several human cancers^1,9,10,12,13,30^, and is one of eight members of a protein family (FAM83A-H) characterized by a highly conserved domain necessary and sufficient to drive transformation^2^. To date, the role of *FAM83B* in thyroid cancers is unclear, though Cipriano and coauthors reported its overexpression in 17 thyroid tumors from a series of different human cancers^1,22^. In the present study, we found a positive correlation between low *FAM83B* mRNA levels and thyroid cell dedifferentiation. Indeed, *FAM83B* was downregulated in TCs and metastatic samples compared to normal thyroid tissues, and concordant data were obtained by the analysis of the TCGA THCA database (https://www.cancer.gov/tcga accessed on May 2020). Consistently, a decrease in *FAM83B* expression was also found in the metastatic tissue with respect to the corresponding primary tumor, which was more evident and significant in distant metastases from FTCs, than in locoregional recurrences from PTCs. Moreover, patients with low *FAM83B* levels tended to have a higher frequency of distant metastatization and a shorter DFS rate than those with high levels, further highlighting the correlation between dedifferentiation and low *FAM83B* mRNA levels, and consistent with data found in ovarian cancer^8^, in lung squamous cell carcinoma—SCC^9^, and triple-negative breast cancer^20^.

Interestingly, we found that *FAM83B* expression levels in TC vary according to the tumor histotype, suggesting the possibility of different roles of this protein in the maintenance and tumorigenicity of the cancer cells. Compared with normal tissues, *FAM83B* resulted constantly and significantly downregulated in PDTCs and ATCs cases. In PTCs a *FAM83B* expression lower than NTs was observed, which was significant comparing paired tissues either from our series or from TCGA. On the other hand, *FAM83B* expression in FTCs was higher than that found in NTs, suggesting that this protein could have a different role in different histotypes, as observed in lung carcinoma^11^.

In order to get more insights into *FAM83B* levels variations in the different thyroid cancer subtypes, we investigated its expression in cell lines derived from healthy and pathological thyroid tissues and we found that they nicely recapitulate the findings obtained in tissue samples. In particular, NTHY-ORI (derived from normal tissue) expressed high levels of *FAM83B* mRNA and protein. Although a variable *FAM83B* expression was found in PTCs, tumors with *RET/PTC1* fusions had levels higher than the median, and this finding was consistent with the high levels recorded in TPC1 cells, which derive from a PTC with a *RET/PTC1* fusion. On the other hand, both SW1736 (derived from ATC) and FTC133 (derived from an FTC metastasis) express low levels, as the corresponding tumor samples. Although the limitations of 2D cell cultures are well known, the variations in FAM83B observed in the different cell lines paralleled those observed both in patients and in the TCGA cohort, thus prompting us to speculate that this model may be useful in the initial understanding of cellular biology of FAM83B in thyroid cancer.

Given that *FAM83B* levels are low in FTC metastatic tissues and in metastasis-derived FTC-133 cell line, we hypothesized a role in cell migration. Several FAM83 family members have been reported to be involved in the regulation of cell migration in different tumors^12,15–19,31^. Interestingly, in the tested cell lines FAM83B protein levels decreased in migrating cells. In particular, NTHY-ORI, which still have contact inhibition but retain migration abilities^23,26^, had high FAM83B levels at confluency, but expression levels promptly decreased when migration was induced by scratch assays. On the other hand, TPC1 showed very low migration abilities, as already reported^24,28^. Consistently, FAM83B expression levels did not vary upon migration induction. Finally, SW1736 and FTC133 cell lines, for which the loss of contact inhibition and the reduction of cell–cell adhesiveness is known^25,27,29^, displayed a further reduction in FAM83B expression during migration. Interestingly, upon migration induction we observed not only a reduction in FAM83B positivity, but also its re-localization from the cytoplasm to the nucleus.

Similar variations were also detected in samples obtained from a FTC patient, with the cytoplasmic FAM83B staining stronger in more differentiated areas and progressively lower in the undifferentiated parts of the tumor. At the metastatic level, the nuclear localization of FAM83B was detected at the invasion front in the firstly developed metastasis and was exclusive and homogeneous in the metastasis developed several years after first treatment. This finding is consistent with what reported for FAM83H in colorectal cancer tissues, where a nuclear staining was observed in a small population of cancer cells located at the edge of or detached from the tumor mass^15^, and indicates that the FAM83 family members, and likely their subcellular localization, might have a role in cell migration as previously suggest in other cancer types^12,15–19,31^.

Finally, results obtained by *FAM83B* silencing on the two cell lines with higher *FAM83B* mRNA levels, NTHY-ORI and TPC1, may support its role in thyroid cancer cell migration. In particular, upon *FAM83B* silencing, migration abilities significantly increased, while the oncogenic RAS/MAPK/PI3K pathways were found not to be affected.

Moreover, we found a decrease of markers of thyroid differentiation and an increase of those associated with acquisition of mesenchymal phenotype, as changes in cell motility are often associated with loss of differentiation or epithelial-to-mesenchymal transition. Although the 2D cell lines model has some intrinsic limitations in the study of cell differentiation, we are tempting to speculate that it can still be considered reliable enough to provide more insight in FAM83B cellular biology. Further studies are needed to confirm FAM83B role in thyroid cell migration and differentiation through 3D multicellular approaches that better resemble patient-derived thyroid tumor.

In conclusion, the whole of the above reported data indicates for the first time a role for FAM83B in thyroid cell differentiation and migration. Since *FAM83B* expression is reduced in more dedifferentiated tumors and in metastases, it is tempting to speculate that it could be involved in the maintenance of a differentiated phenotype. In dedifferentiated cells, its decreased expression and its nuclear re-localization could favour distant migration. A low *FAM83B* expression, even if limited to a part of a tumour sample, could thus indicate the possible existence of circulating tumour cells eventually responsible for distant metastases. For these reasons, *FAM83B* should be considered a possible diagnostic and prognostic biomarker.

## Materials and methods

### Patients and samples collect

This is a retrospective cohort study with institutional review board approval (Ethical Committee of the Istituto Auxologico Italiano IRCCS, #2018_09_25_04). Informed consent for the use of thyroid tumor tissues and collection of clinico-pathological information was obtained from all participants. All methods were carried out in accordance with relevant guidelines and regulations (Declaration of Helsinki).

Frozen thyroid tissues from 53 patients undergone total thyroidectomy were used for molecular and expression analysis. In particular, we studied 20 papillary (PTC), 5 follicular thyroid cancers (FTC), 9 poorly differentiated (PDTCs)/anaplastic thyroid cancers (ATC), 16 metastases (MTS), 24 normal thyroid tissues (NT). Formalin-fixed paraffin embedded (FFPE) thyroid tissues were obtained for immunohistochemistry analyses. Patients are followed in a single tertiary care endocrine center and were diagnosed and treated according to the recent guidelines for the management of thyroid cancer during the period 2001–2020^32,33^. Tumors were classified and staged according to the thyroid malignancy World Health Organization classification and the 8th edition of TNM staging^34^. Clinico-pathological features at diagnosis and the final disease outcome, after a mean follow-up of 37 months (range 8–219), were available for all included patients. The molecular characterization of all PTC cases was done by means of a fully validated custom PTC-MA assay, based on matrix-assisted laser desorption/ionization time-of-flight mass spectrometry^35,36^.

### Cell culture and siRNA transfection

NTHY-ORI 3-1 (immortalized normal thyroid), TPC1 (derived from human papillary thyroid carcinoma with RET/PTC1 fusion) and K1 (derived from a papillary thyroid cancer with BRAFV600E mutation) cells were grown in RPMI supplemented with Glutamine (Euroclone, Pero, Italy), SW1736 (derived from human anaplastic thyroid carcinoma) cells, SW579 cells (derived from a poorly differentiated carcinoma) and HTCC3 cells (derived from a pleural metastasis of poorly differentiated thyroid carcinoma) were grown in DMEM (Gibco-Thermo-Fisher Scientific), while FTC133 cells (derived from a lymph node metastasis of human follicular thyroid carcinoma) were grown in DMEM/F12 (Gibco-Thermo-Fisher Scientific) in a humidified incubator at 37 °C under 5% CO_2_. All media were supplemented with 10% fetal bovine serum (SigmaAldrich, ST. Louis, Missouri, USA) and penicillin–streptomycin mixture (Sigma-Aldrich). *FAM83B* expression was downregulated by small interfering RNA (siRNA). Two siRNAs against *FAM83B* (Hs_FAM83B_8 and Hs_FAM83B_9 FlexiTube siRNAs, Qiagen GmbH, Hilden, Germany)^11^ and a control one (Negative Control FlexiTube siRNA, Qiagen) were transfected in 6-well dishes to a final concentration of 10 nM with HiPerFect (Quiagen) following manufacturer’s instruction. All experiments were performed 3 days after transfection. All experiments were performed with cell lines in between the 7th and the 15th passage. All cell lines were authenticated by short tandem repeat (STR) profiling and were routinely screened for mycoplasma contamination with Venor GeM Classic Mycoplasma Detection Kit (Minerva Biolabs GMBH, Berlino, Germany).

### Quantitative real-time PCR (qRT-PCR)

Total RNA was isolated from frozen thyroid tissues and thyroid healthy and tumor cell lines using Trizol reagent (Thermo Fisher Scientific, Waltham, Massachusetts, USA) according to the manufactures’ instructions. RNA was quantified at spectrophotometry, and its quality was checked by the analysis of 260/280 nm and 260/230 nm ratios. 1000 ng of each RNA sample was reverse transcribed using a Superscript reverse transcriptase II Kit (ThermoFisher Scientific), with random hexamers mixture as primers. Appropriated endogenous controls for qRT-PCR were selected with Applied Biosystems^®^ TaqMan^®^ Express Endogenous Control Plates (ThermoFisher Scientific) among 32 constitutively expressed different genes tested on healthy and pathological tissues. Among the 32 candidate genes, two genes (*ACTB* and *B2M*) were indicated as the most reliable (appropriate and similar cycle threshold –Ct– values between tumor and normal thyroid tissue samples), and were thus selected as endogenous controls for all experiments, as optimal balance between analysis accuracy and samples mRNA availability. RNA was analysed for *FAM83B*, *ACTB*, *B2M* expression using an Applied Biosystems QuantStudio 12K Flex Real-Time PCR System (Thermofisher Scientific) and the following assay Hs00289694_m1, Hs99999907_m1 and Hs99999903_m1 (Thermofisher). Relative *FAM83B* expression levels were calculated using the 2-ΔΔCt method. The geometric mean of Ct values was used as averaging method of the control genes, as previously reported^37,38^. For each patient the retro-transcription and Real-Time PCR were repeated at least three times, with each sample run in triplicate. A pool of 20 healthy thyroid tissues (10 from women and 10 from men) was run in each experiment together with the samples, and used as reference control.

For RNA of migrating cells, confluent cells seeded in 6-well plates were scratched with a p1000 tip multiple times to obtain alternating confluent cells and free space areas. After 24 h, when most of the cells were moving through the free spaces, samples were lysed together with unscratched samples as control.

### Gene expression analysis by semiquantitative RT-PCR

Total RNA was extracted from NTHY-ORI cells non transfected (NT) or transfected with siRNA scramble as control (siCT) and against FAM83B (siFAM 1 and siFAM 2) using Trizol reagent (Thermo Fisher Scientific, Waltham, Massachusetts, USA) according to the manufactures’ instructions. 1000 ng of each RNA sample was reverse transcribed using a Superscript reverse transcriptase II Kit (ThermoFisher Scientific), with random hexamers mixture as primers. *PAX8* and *NIS* mRNA were amplified in the log-linear phase of PCR by 25–34 cycles using 100 ng of each cDNA and primers previously reported^39^. In all PCR analyses, the amount of cDNA in each sample was normalized using *ACTB* as an internal control. The PCR products were separated by electrophoresis on 2% agarose gels stained with Midori Green Advance DNA Stain (Nippon Genetics Europe GmbH, Düren, Germany). The signal intensities of gene bands were analyzed with Image J software (version 2.1.0/1.53c; National Institutes of Health, Bethesda, MD). Full-length agarose gel images are shown in Supplementary Fig. 5.

### Immunohistochemistry (IHC)

Immunohistochemical studies were carried out on one out of four consecutive 4 µm thick sections obtained from paraffin blocks. According to the manufacturer’s instructions, tissue sections were incubated overnight with 1:200 dilution of anti-FAM83B antibody (rabbit polyclonal IgG antibody, Atlas Antibodies, Voltavägen, Bromma, Sweden). Slides were evaluated independently by two experts (G.G., L.E.). Image acquisition was performed by LEICA ICC 50 HD.

### Wound healing and migration assays

For wound healing assay, samples were processed as previously described^40^. Confluent cells seeded in 6-wells plates were scratched once with a p200 tip with a single vertical movement, washed with PBS and returned to the appropriated medium. At the indicated times post-wound, cells were washed with PBS, fixed with 2% PFA in PBS for 20 min RT and stained with 0.01% Crystal Violet PBS solution for 30 min RT. The samples were washed 3 times in H2O and then let air-dry overnight. Images were acquired basally and at 16 h post-wound with a Samsung J510FN camera on Olympus CK2 microscope with A10PL 10× objective. Wound dimensions were quantified with FIJI^41^. For western blot of migrating cells, confluent cells seeded in 6-well plates were scratched with a p1000 tip multiple times to obtain alternating confluent cells and free space areas. After 24 h, when most of the cells were moving through the free spaces, samples were lysed together with control unscratched samples, as described in “Western blotting” section. For immunofluorescence of migrating cells, confluent cells seeded onto poly-l-lysine (Sigma-Aldrich) coated microscope slides in 6-well plates were gently scratched with a p200 tip. After 6 h, scratched samples together with control unscratched samples, were processed as described in “Immunofluorescence” section.

### Western blotting

Cells were lysed in SDS sample buffer (62.5 mM Tris–HCl pH 6.8, 2% sodium dodecyl sulfate) supplemented with protease, phosphatase and proteasome inhibitors, pre-heated at 95 °C^40^. Thereafter, samples were sonicated and protein amount dosed with Pierce BCA protein Assay Kit (Thermo Fisher Scientific). Sixty µg of protein extracts were then separated on NuPage 4–12% Bis–Tris Gels (Thermo Fisher Scientific) and transferred with iBlot System (Thermo Fisher Scientific). Membranes were incubated with blocking buffer (5% nonfat dry milk in TBS-T solution) for 1 h at room temperature and probed overnight at 4 °C with appropriate primary antibodies diluted 1:1000 in blocking buffer: antiFAN83B (Atlas Antiboides), anti-vimentin and anti-SDHA (Abcam), anti-p-ERK1/2, anti-ERK1/2, anti-P-AKT and anti-AKT (Cell Signalling), as previously described^40^. After washing, membranes were incubated for 1 h at room temperature in the presence of the appropriated HRP-conjugated secondary antibody (Merck Millipore, Burlington, Massachusetts, USA) diluted to 1:5000 in blocking buffer. Detection was performed utilizing Westar Supernova ECL Substrate (Cyanagen, Bologna, Italy) and images acquired with c400 camera (Azure Biosystems, Sierra Ct, Dublin, USA). Band intensity was quantified with FIJI software^41^. Full-length blot images are shown in Supplementary Figs. 4–8.

### Immunofluorescence

Samples were washed with pre-warmed PBS and incubated in the dark for 5 min with Whole Germ Agglutinin (WGA) Alexa-Fluor 594 conjugated (Thermo Fisher Scientific) 5 µg/µl solution in pre-warmed PBS at 37 °C in 5% CO2. After washing in PBS, samples fixed by incubation in pre-warmed 4% PFA in PBS solution for 10 min. After washing with PBS, cells were permeabilized with 0.1% saponin in PBS for 5 min and then blocked with 5% BSA in PBS at room temperature for 1 h. Samples were incubated overnight at 4 °C with the anti-FAM83B primary antibody above reported, diluted to 1:100 in blocking buffer. On the following day, cells were washed three times in PBS, and 1-h incubation was performed with appropriated secondary antibody solution diluted to 1:500 in blocking buffer. Samples were mounted on microscope slides with 20 μl of Vectashield Hard-Set mounting medium with DAPI (Vector Laboratories, Burlingame, California, USA). Whole cells were acquired on the basis of WGA staining with Z-series acquisition, 0.15 μm steps with Nikon EclipseTi-E inverted microscope equipped with Intensilight C-HGFIE Epi-fluorescence illuminator Long-life mercury light source; all images were acquired with Nikon Plan Apo λ 100× objective. The total number of cells analyzed was 110 for NTHY-ORI 3-1 (57 confluent and 53 migrating), 136 for TPC1 (66 migrating and 70 confluent), 135 for SW1736 (64 confluent and 71 migrating), 97 for FTC133 (56 confluent and 41 migrating), for a total of 4 independent replicates for each cell line.

### Bioinformatic analysis

Gene expression RNAseq Data from The Cancer Genome Atlas (TCGA, RRID:SCR_003193) (https://www.cancer.gov/tcga, accessed on May 2020) THCA dataset^42^ were processed with UCSC Xena^43^ as of September 2020. The .tsv files with data expressed as log2 (norm_count + 1) were downloaded and further analyzed with Graphpad Prism 5.

### Statistical analysis

For the analysis of *FAM83B* mRNA levels, the geometric mean of Ct values of the 2 best identified endogenous control was used as averaging method, as previously reported^37,38^. In all groups, the *FAM83B* mRNA values showed a non-normal distribution, with a positive skewed distribution in Normal Thyroid (NT) and Thyroid Cancer (TC) groups. Given the skewed distribution of most groups and the presence of outliers, the data were represented as median and interquartile range. No bimodal distribution of *FAM83B* values was detected. For Kaplan-Meyer survival curves, patients were divided in *FAM83B*-high and *FAM83B*-low based on *FAM83B* TC group median value (0.517). Patients with *FAM83B* values within 10 percentiles from median (0.482–0.607) were excluded from the analysis (n = 7). For *FAM83B* gene expression, data were available for 505/507 patients from The Cancer Genome Atlas (TCGA, RRID:SCR_003193) (https://www.cancer.gov/tcga accessed on May 2020) THCA dataset^40^ (Thyroid Cancer (TC) n = 505; paired FAM83B levels available for Normal Thyroid (NT) n = 58 and Metastasis (MTS) n = 8). In order to validate whether *FAM83B* expression could predict prognosis in thyroid cancer patients, Kaplan–Meier analysis and Log-rank test were used to assess the association between *FAM83B* expression and disease-specific survival rate.

For immunofluorescence experiments, co-localization was measured by Pearson’s R coefficient in 4 independent experiments with FIJI Coloc2 plugin^39^. For FAM83B intensity quantification, Raw Integrated Density was measured with FIJI after determination of Regions Of Interest (ROIs) based on WGA for total FAM83B, DAPI for nuclear FAM83B, and WGA minus DAPI for cytoplasmic FAM83B.

After normal distribution and variance similarity evaluation, Kruskal–Wallis test followed by Dunn's Multiple Comparison Test, Wilcoxon signed rank test, and Mann Whitney test were used to determine statistical significance with Graphpad Prism 5 (RRID:SCR_002798), as indicated in the corresponding Figure legends. Significance expressed as p values (*p < 0.05, **p < 0.01, ***p < 0.001).

## Supplementary Information

Supplementary Table 1.

Supplementary Figures.
