# Supplementary material for: FAM83B is involved in thyroid cancer cell differentiation and migration
Source: Sci Rep. 2022 May 21;12:8608. doi: 10.1038/s41598-022-12553-2 (PMC9124208; doi:10.1038/s41598-022-12553-2)
Supplement: Supplementary file 1 — Supplementary Table 1. [file 41598_2022_12553_MOESM1_ESM.docx]

**Supplemental Table 1**: clinico-pathological characteristics of the tumors included, divided according *FAM83B* levels. Patients were divided in *FAM83B*-high and *FAM83B*-low based on *FAM83B* thyroid cancer group median value (0.517). Patients with *FAM83B* values within 10 percentiles from median (0.482-0.607) were excluded from the analysis (n=7).

|  | **FAM83B HIGH**  (n = 14) | **FAM83B LOW**  (n =13) | **P** |
| --- | --- | --- | --- |
| Histotype, PTC/FTC/PDTC-ATC | 8/5/1 (57/36/7%) | 8/0/5 (62/0/38%) | **0.021** |
| Gender, female | 9 (64%) | 7 (64%) | 0.588 |
| Mean age at diagnosis, yrs ± SD (range) | 60.1 ± 17.7 (25-81) | 49.4 ± 22.6 (19-87) | 0.259 |
| Presence of extrathyroidal extension | 5 (36%) | 7 (54%) | 0.352 |
| Presence of lymphnode metastases | 7 (50%) | 2 (15%) | 0.061 |
| Presence of distant metastases | 3 (21%) | 6 (64%) | 0.181 |
| Mean tumor size, mm ± SD (range) | 37.6 ± 2.6 (17-95) | 36.9 ± 2.4 (15-90) | 0.942 |
| Persistence at last follow-up | 6 (43%) | 8 (62%) | 0.340 |

Legend: PTC, Papillary Thyroid Carcinoma; FCT, Follicular Thyroid Carcinoma; PDTC, Poorly Differentiated Thyroid Carcinoma; ATC, Anaplastic Thyroid Carcinoma; SD; Standard Deviation
