# Supplementary material for: FAM83B is involved in thyroid cancer cell differentiation and migration
Source: Sci Rep. 2022 May 21;12:8608. doi: 10.1038/s41598-022-12553-2 (PMC9124208; doi:10.1038/s41598-022-12553-2)

**Supplementary Figure 1: FAM83B expression in thyroid paraffin-embedded tissues.**

A positive cytoplasmatic FAM83B staining was observed in FFPE normal thyroid tissue (A) and in follicular thyroid carcinoma (B) samples (Magnification 40X). No FAM83B staining was detected in papillary thyroid carcinoma (C) and in poorly differentiated thyroid carcinoma (D) samples (Magnification 40X).

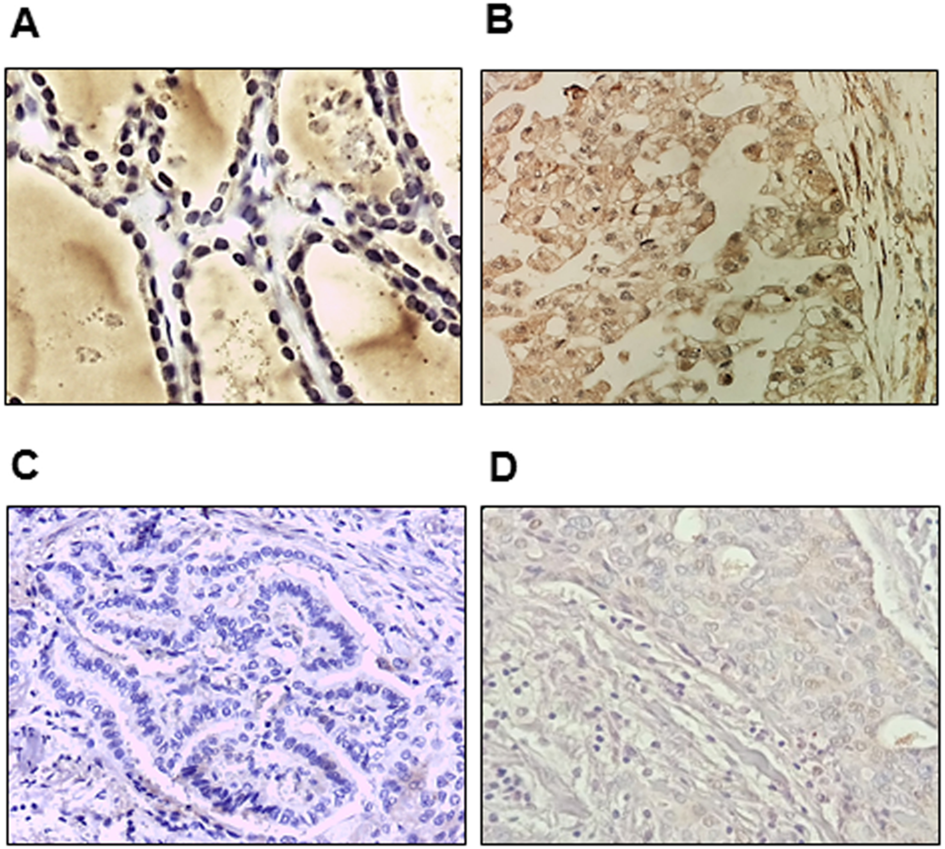

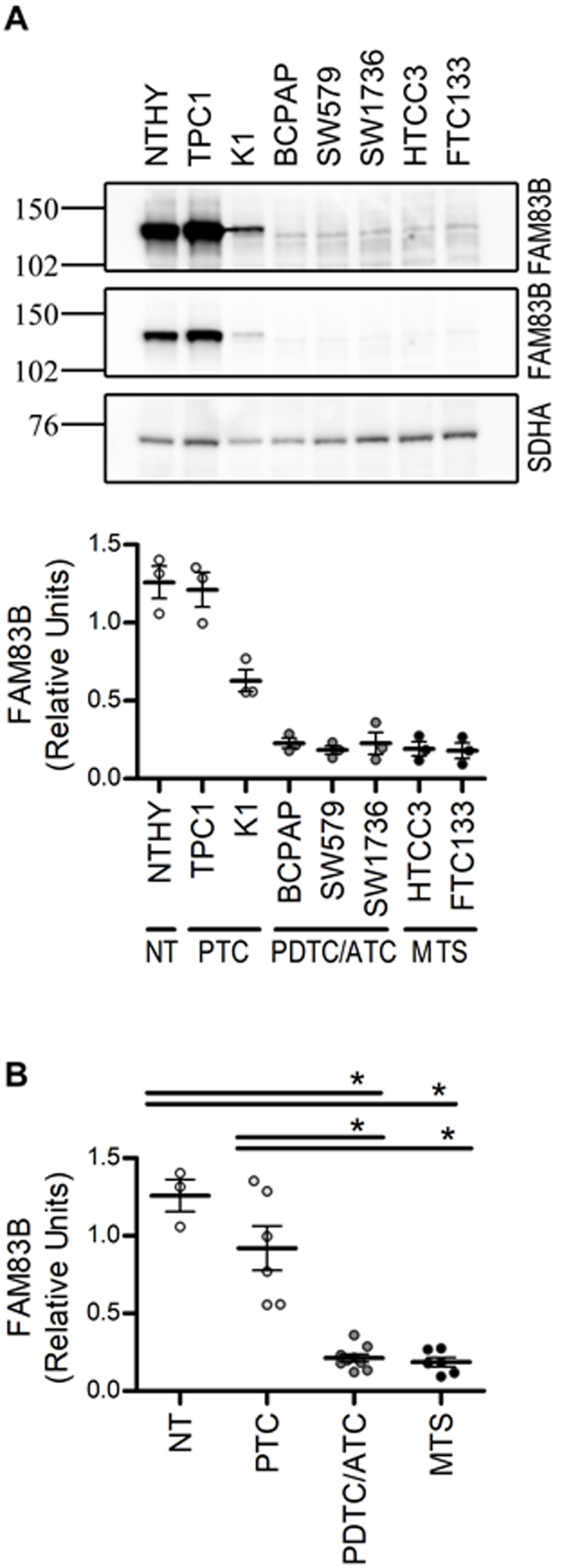


**Supplementary Figure 2: FAM83B levels in different thyroid cancer cell lines.**
**Panel A:** Representative images and densitometric analysis of western blots showing FAM83B levels in eight different normal and cancer thyroid cell lines. Two different exposure times are presented to better appreciate the differences among high-FAM and low-FAM expressing cells. Full-length blot images are shown in **Supplementary Figure 8**.
**Panel B:** FAM83B densitometric quantification of the eight cell lines grouped by tissue of origins.
*Statistical analysis*: **A-B**, Kruskal-Wallis test followed by Dunn's Multiple Comparison Test.

**Supplementary Figure 3: full FAM83B immunofluorescence images.**
Representative immunofluorescence images with all channels used for Figure 5 analysis of confluent (Panel A) and migrating (Panel B) cells. DAPI was used to identify nuclear ROI while WGA was used to identify whole cell ROI. Scalebars 10 µm.

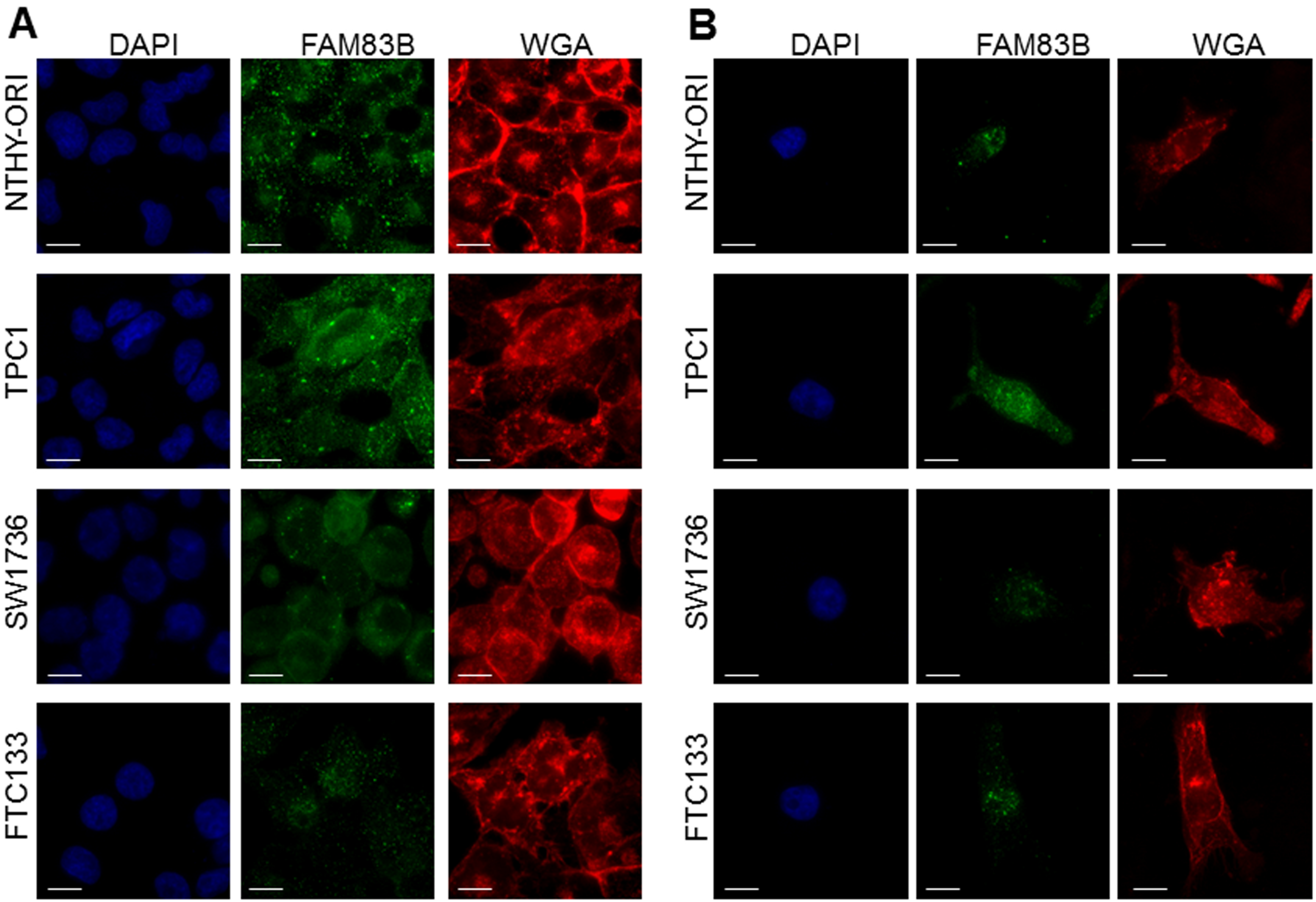

**Supplementary Figure 4:**
**full-length blots images.**
Left panel: full-length blots of Figure 3B representative images.
Right panel: full-length blots of Figure 3E representative images.
The regions of these original blots used in main figures are highlighted with red boxes.

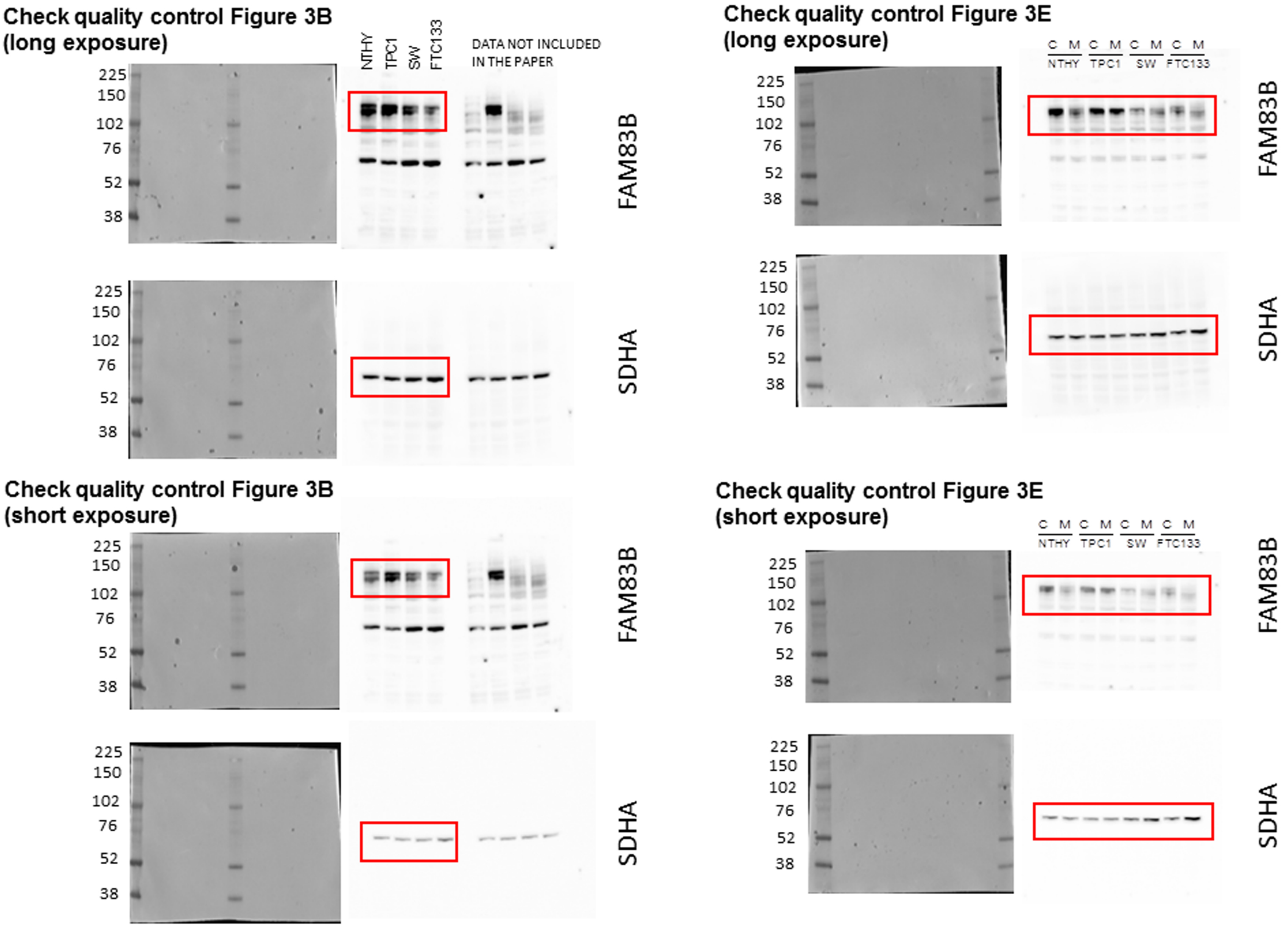

**Supplementary Figure 5: full-length-blots images.**
**Left panel:** full-length blots of Figure 6A representative images.
**Middle panel:** full-length agarose gel of Figure 6B representative images.
**Right panel:** full-length blots of Figure 6C representative images.
The regions of these original blots used in main figures are highlighted with red boxes.

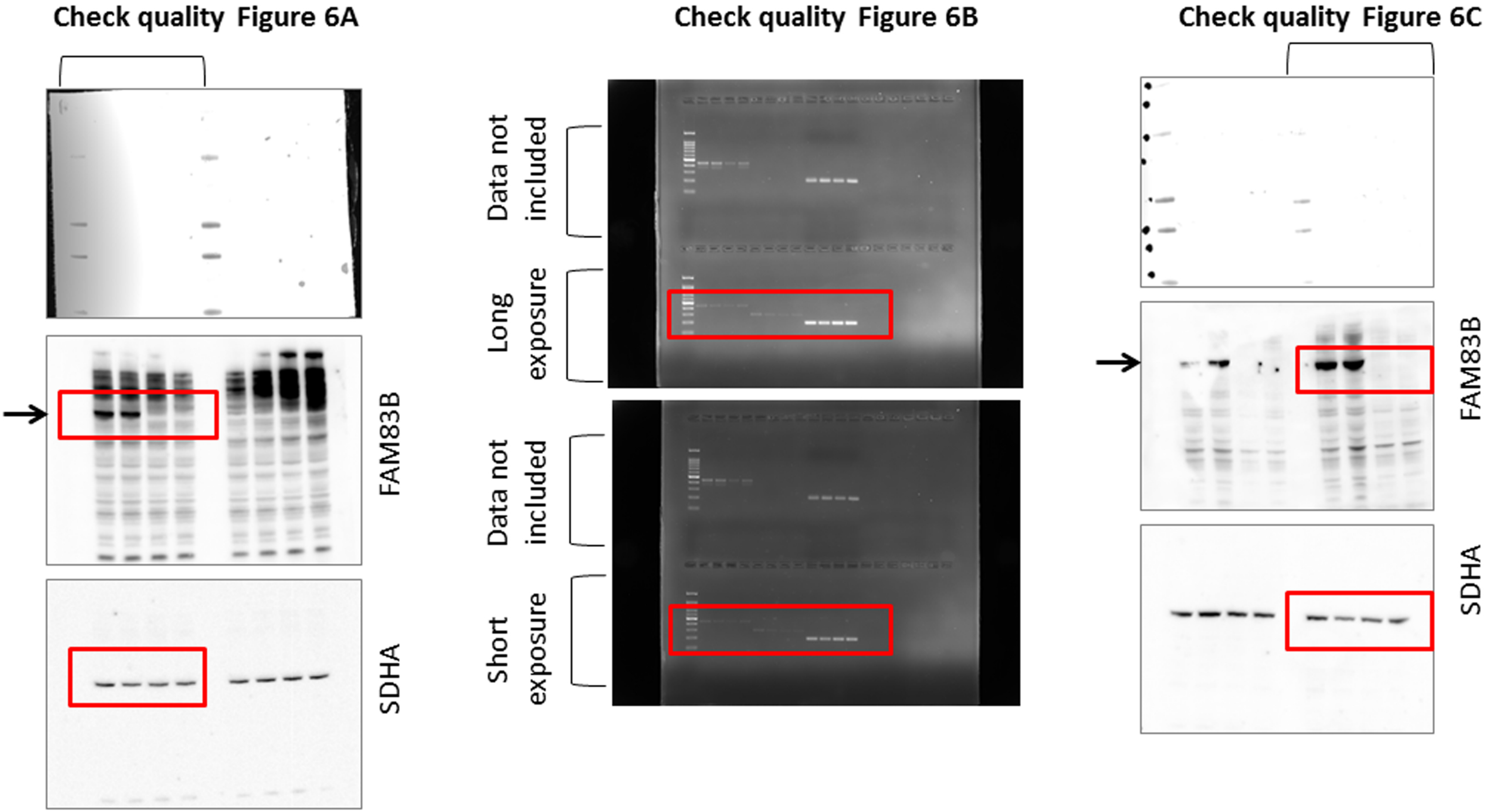

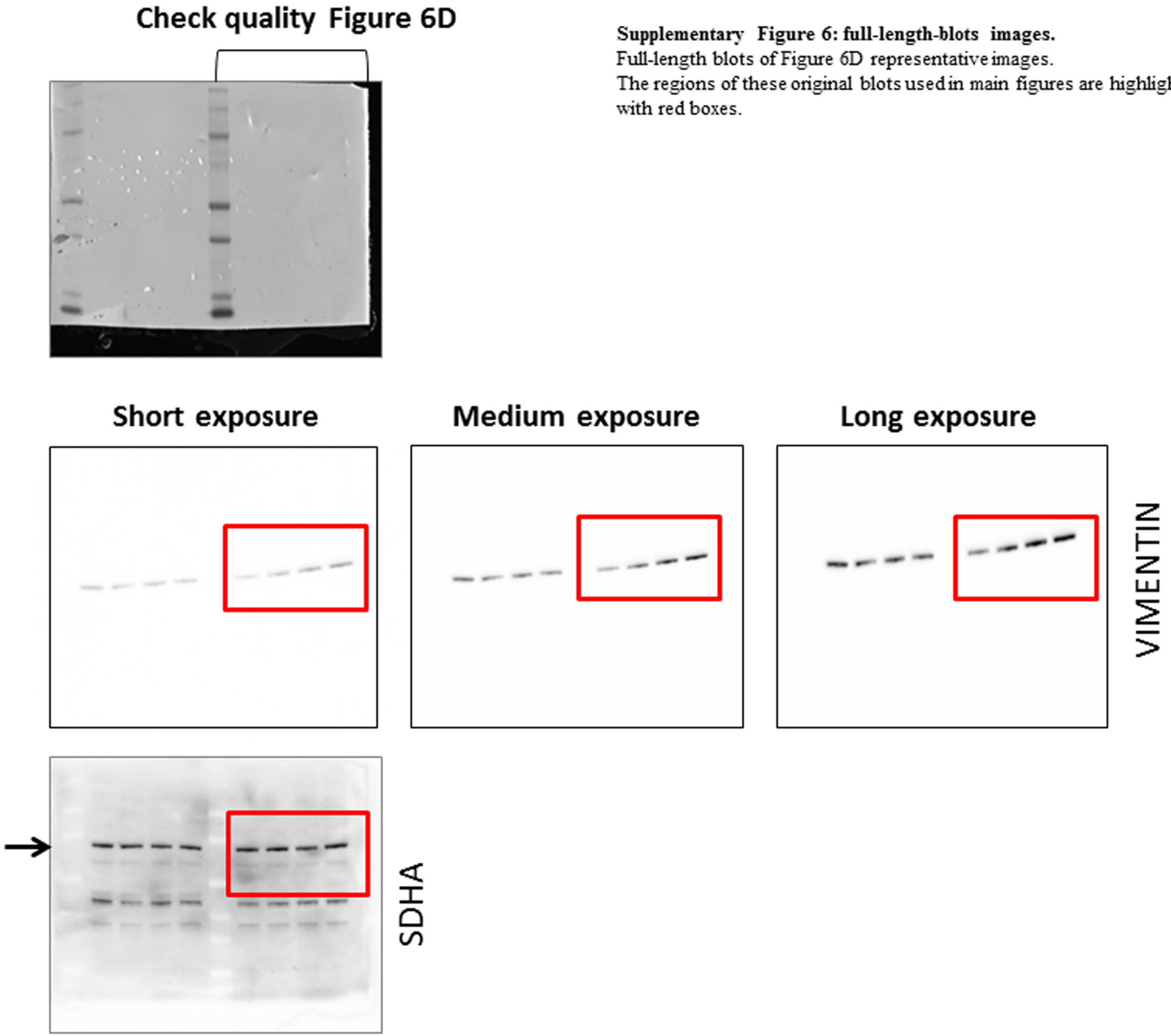


**Supplementary Figure 6: full-length-blots images.**
Full-length blots of Figure 6D representative images.
The regions of these original blots used in main figures are highlighted with red boxes.

**Supplementary Figure 7: full-length-blots images.**
Full-length blots of Figure 6E representative images.
The regions of these original blots used in main figures are highlighted with red boxes.

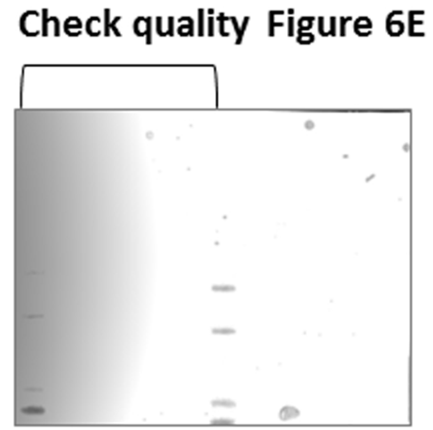


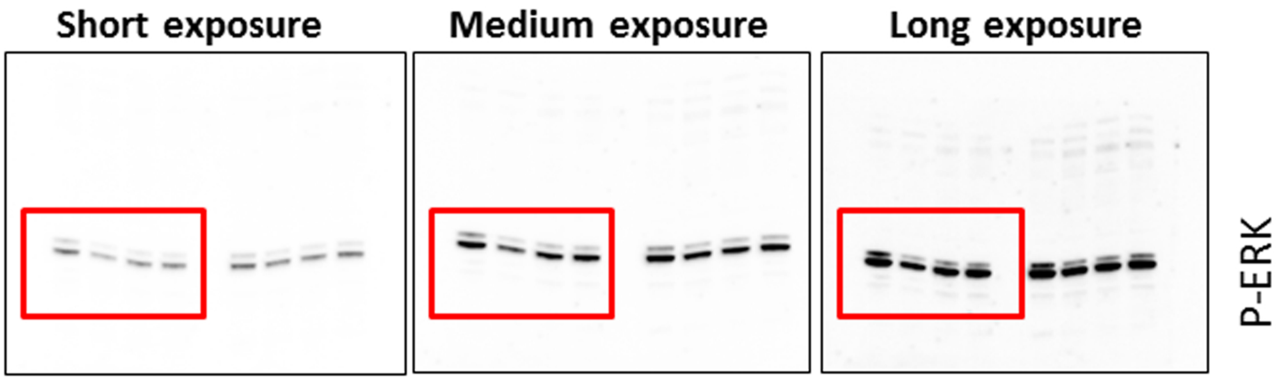


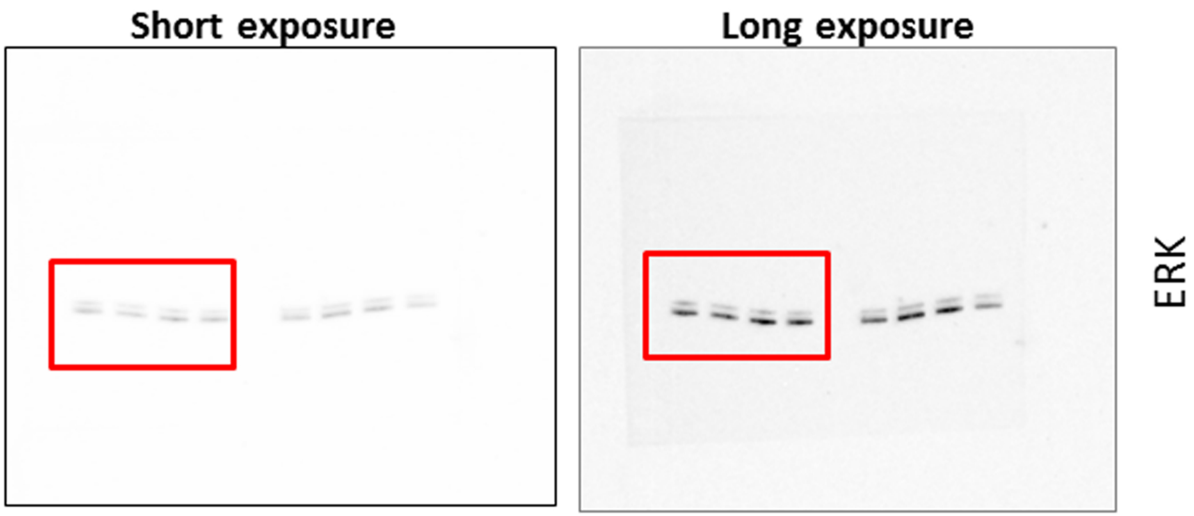


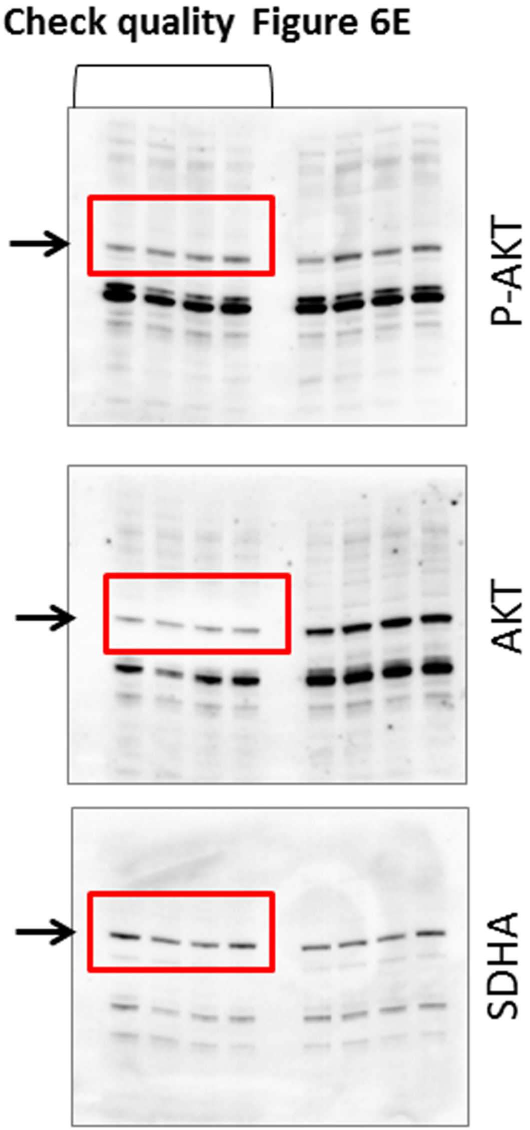

**Supplementary Figure 8: full-length-blots images.**
Full-length blots of Supplementary Figure 2A representative images.
The regions of these original blots used in main figures are highlighted with red boxes.

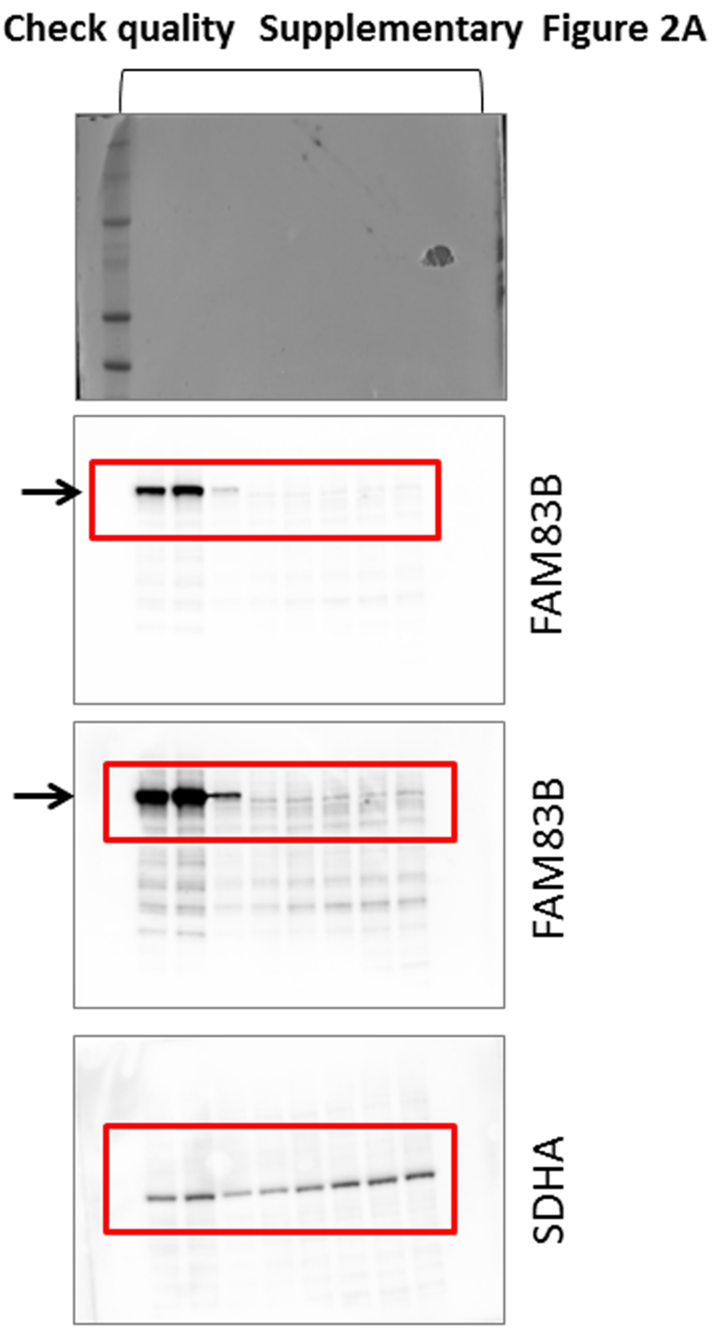

Supplement: Supplementary file 2 — Supplementary Figures. [file 41598_2022_12553_MOESM2_ESM.pdf]
